# In Vitro and In Vivo Characterization of PCC0104005, a Novel Modulator of Serotonin-Dopamine Activity, as an Atypical Antipsychotic Drug

**DOI:** 10.1038/s41598-018-25036-0

**Published:** 2018-05-02

**Authors:** Yanan Xu, Xiaoyin Zhu, Hongbo Wang, Shanyue Sun, Xin Yue, Jingwei Tian

**Affiliations:** School of Pharmacy, Key Laboratory of Molecular Pharmacology and Drug Evaluation (Yantai University), Ministry of Education, Collaborative Innovation Center of Advanced Drug Delivery System and Biotech Drugs in Universities of Shandong, Yantai University, Yantai, 264005 P.R. China

## Abstract

PCC0104005 is a novel drug candidate for treating schizophrenia that displays high affinity for serotonin, dopamine, and noradrenaline receptors, including partial agonism at dopamine D_2_, D_3_, D_4_, serotonin 5-HT_1A_, and 5-HT_2A_ receptors and antagonism at 5-HT_2B_, 5-HT_6_, and 5-HT_7_ receptors. PCC0104005 blocks MK-801-induced hyperactivity in rats, consistent with the reduction in dopamine D_2_ receptor stimulation and increased dopamine release in the medial prefrontal cortex. PCC0104005 inhibits 5-HTP-induced head twitches in rats, due to its moderate affinity for human 5-HT_2A_ receptors (Ki = 5.1 nM). PCC0104005 significantly reduced the escape latency of rats and improved the MK-801-induced memory impairment. In the object recognition experiment, PCC0104005 significantly improved the recognition disorder induced by MK-801. PCC0104005 did not significantly increase the plasma prolactin level, which is thought to be related to the preferential affinity of PCC0104005 for dopamine D_2_ receptors compared with 5-HT_1A_ receptors, as well as the relative antagonistic activity toward the D_2_ receptor. Due to its 5-HT_1A_ agonism, PCC0104005 does not produce catalepsy in mice, a behaviour predictive of the occurrence of extra-pyramidal syndrome (EPS) in humans. PCC0104005 has unique affinities for dopamine receptors and serotonin receptors, which may lead to clinical advantages, as well as fewer adverse reactions.

## Introduction

Schizophrenia is a major neuropsychiatric disorder that affects more than 1% of the global population. It is characterized by hallucinations and delusions of experience, known as “positive” symptoms, as well as various other symptoms, including reduced social function and speech, emotional deprivation, the confusion of thought, and low motivation, known as “negative” symptoms. Cognitive dysfunction is also a central feature of schizophrenia^1^. The treatment of schizophrenia, now known as “typical” antipsychotic drugs that exert a common strong inhibitory effect on the D_2_ class of dopamine receptors, has undergone a revolutionary change since the first generation of antipsychotic drugs were developed 60 years ago^2^.

The use of a typical antipsychotic medication is an effective way to reduce the positive symptoms in many patients. Because of its potent D_2_ receptor antagonism in the substantia nigra striatum in the motor system, its utility is limited by severe side effects, including acute Parkinson’s disease disorders and dystonia, commonly known as extrapyramidal symptoms (EPS), and drug-induced delayed dyskinesia^3–5^.

One of the key issues in the excitatory action of D_2_ is to determine its optimal level of intrinsic activity. When the intrinsic activity of the drug at the D2 receptor is too high, a lack of potent clinical activity and a greater number side effects are observed that increase the tension of D_2_ receptors, including nausea, vomiting, insomnia, and motor effects^6^, whereas excessive D_2_ antagonist activity results in an increased risk of EPS by increasing prolactin secretion^7^. It has been reported that antipsychotic responses and extrapyramidal side effects (EPS) may be related to D_2_ receptor occupancy, albeit with different thresholds^8^. With typical antipsychotics, a D_2_ receptor occupancy in the range of 70% was associated with the clinical response, whereas EPS emerged at a D_2_ receptor occupancy of 80%^8^. The main strategy for treating schizophrenia is based on antagonizing dopamine D_2_ receptors. However, due to the problem of tolerance, treatment with D_2_ receptor antagonists is not considered the optimal strategy for regulating dopaminergic activity, and the discovery and development of D_2_ receptor partial agonists provides stable dopamine function in an affordable treatment. In addition, most second-generation antipsychotics are serotonin 5-HT_2A_ receptor and adrenergic α_1_ receptor antagonists, and individual componds have an effect on a variety of other monoamine receptors, such as the 5-HT_1A_ receptor. These broad target effects also improve the efficacy of antipsychotic drugs (with additional effects on emotional symptoms or cognitive disorders) or mitigate adverse effects [e.g., EPS]^9,10^.

Multiple evidences suggest that schizophrenia may be associated with glutamate dysfunction^11,12^. NMDA receptor antagonists can aggravate symptoms in patients with schizophrenia^13,14^. In rats, the highly selective non-competitive NMDA receptor antagonist, MK-801, induces hyperlocomotion and other signs of disorganized behaviour^15^. These findings have led to the use of MK-801-treated rodents as models for schizophrenia1. Although the drug-based acute effects do not contain developmental components when applied to adult animals, however, this model shows good predictive validity, and importantly, it triggers many symptoms similar to those in Symptoms observed in affected human subjects^16^.

A new antipsychotic medication acting synergistically via serotonergic, dopaminergic, and glutamatergic receptors that is able to improve the social functioning of patients while addressing positive symptoms of the disease with a lower incidence of EPS would be of tremendous benefit to patients with schizophrenia. Here, we describe the biochemical and behavioural characterization of PCC0104005, a novel small-molecule therapeutic agent displaying the combined properties of dopamine D_2_ receptor antagonism and serotonin transporter binding, which is currently in development for the treatment of schizophrenia. The findings provide a new drug option for antipsychotic treatment.

## Results

### Receptor binding profile analysis

**Figure 1:**
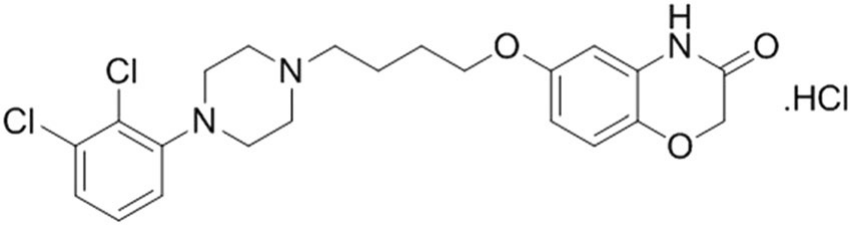
The structure of PCC0104005.

The structure of PCC0104005, a hydrochloric acid, is shown in Fig. 1. Binding affinities of the compound for receptors implicated in the therapeutic actions of antipsychotic medications, including serotonin receptors, dopamine receptors, adrenergic receptors and histamine H_1_ receptors are shown in Table 1. PCC0104005 displays high-affinity binding to the D_2_ receptors, with a Ki = 0.11 nM, and the D_3_ receptors, with a Ki = 0.14 nM. Aripiprazole has higher affinities for 5-HT_2A_ receptors than PCC0104005 (Ki = 3.4 nM and 5.1 nM, respectively). PCC0104005 possesses a higher selectivity for D_2_ receptors than 5-HT_2A_ receptors. When the antipsychotic medications are adrenergic 5-HT_2C_ receptor and histamine H_1_ receptor antagonists, it is more likely to cause adverse reactions.However, PCC0104005 also displayed high affinity for H_1_ receptors, with a Ki = 1.1 nM, that may cause EPS and other adverse reactions. Compared to risperidone, PCC0104005 displayed moderate affinity for 5-HT_2C_ receptors (Ki = 12 nM and Ki = 36 nM, respectively), which is conducive to reducing abnormal glucose and lipid metabolism, weight gain and other adverse reactions.

**Table 1:** Receptor binding affinity of PCC0104005, as measured by radioligand displacement assays: comparison with antipsychotic medications.

Drugs	D_2_	D_3_	D_4_	5-HT_1A_	5-HT_2A_	5-HT_2B_	5-HT_2C_	5-HT_6_	5-HT_7_	α_1A_	α_1B_	H_1_
PCC0104005^a^	0.11	0.14	24	—	5.1	0.34	36	85	5	8.3	3.2	1.1
Aripiprazole^b^	0.34	0.8	44	1.7	3.4	0.4	15	—	39	57	—	61
Risperidone^b^	3.57	14.7	4.66	423	0.17	61.9	12	—	6.6	3	—	60

^a^PCC0104005 affinities were determined as described in the “***Materials and Methods***” section.

^b^Binding affinities for other compounds were derived from Wikipedia.

### Reversal of MK-801-induced locomotor hyperactivity in rats

**Figure 2:**
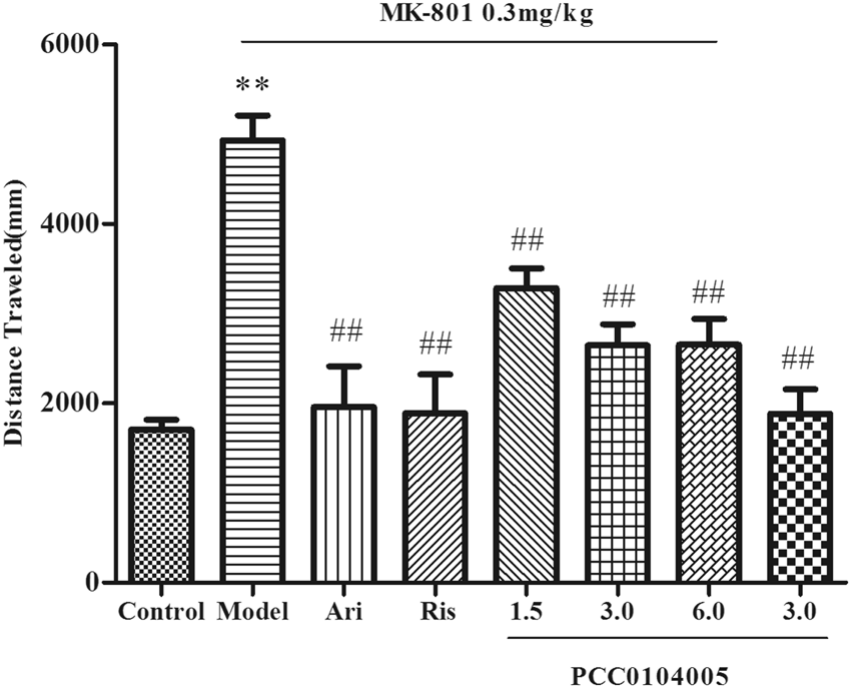
The effect of PCC0104005 on MK-801-induced hyperlocomotion in rats. Rats were administered an oral dose of vehicle alone (0.5% methylcellulose, 1 ml/kg), PCC0104005 (1.5, 3.0, or 6.0 mg/kg), aripiprazole (1.5 mg/kg) or risperidone (0.2 mg/kg) 1 h before test. Then, all animals were intraperitoneally injected with 0.9% saline or MK-801(0.3 mg/kg) 10 minutes before test. Locomotor activity was recorded for 10–20 minutes after the intraperitoneal injection. Results are presented as mean ± S.E.M. (n = 8). *P < 0.05, **P < 0.01 model group vs. the control group, ^#^P < 0.05, ^##^P < 0.01 drug delivery groups vs. the model group.

The MK-801 treatment gradually increased activity and reached a stable level after approximately 10 minutes (distance travelled = 4933 ± 275.6 mm), thus maintaining stability for at least 60 minutes after injection. Aripiprazole and risperidone significantly reduced MK-801-induced hyperactivity in rats (distance travelled = 1957 ± 455.1 mm, F(1,14) = 47.124, P = 0.000 and distance travelled = 1886 ± 437.3 mm, F(1,14) = 52.954, P = 0.000, respectively). All three doses of PCC0104005 significantly inhibited the locomotor hyperactivity induced by MK-801 in rats (distance travelled = 3284 ± 220.7 mm, P = 0.004, 2646 ± 235.2 mm, P = 0.000, and 2655 ± 286.5 mm, P = 0.000, respectively, F(3.28) = 23.829, P < 0.01). The distance travelled by rats in the 3.0 mg/kg and 6.0 mg/kg PCC0104005 groups was similar to the aripiprazole and risperidone groups (F(3.28) = 3.110, P > 0.05) (Fig. 2). When administered alone, PCC0104005 had no significant effect on spontaneous activity in rats (distance travelled = 1885 ± 269.7 mm). Other stereotypic behaviours, such as licking or grooming, were the same as saline-treated animals upon a visual examination (during the 20-min period following MK-801 administration).

### Head twitch behaviour induced by 5-HTP in rats

**Figure 3:**
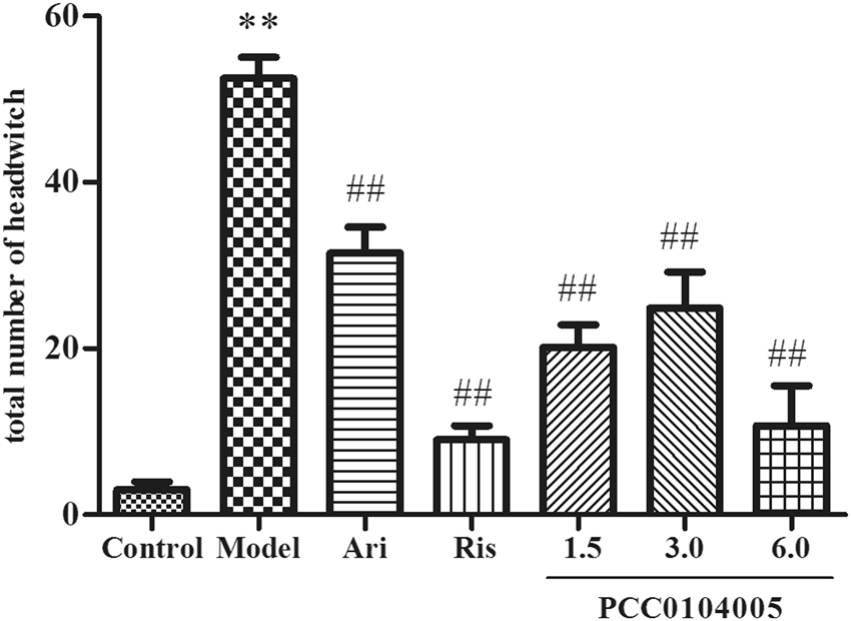
The effect of PCC0104005 on 5-HTP induced head twitches within 30 minutes. Rats were administered an oral dose of vehicle alone (0.5% methylcellulose, 1 ml/kg), PCC0104005 (1.5, 3.0, or 6.0 mg/kg), aripiprazole (Ari, 1.5 mg/kg) or risperidone (Ris, 0.2 mg/kg) was orally administered 1 hour before the experiment. Twenty minutes later, 0.9% saline or 5-HTP (320 mg/kg) was intraperitoneally injected, and the total number of head twitches was recorded during a period of 40–70 min after intraperitoneal injection. Results are presented as mean ± S.E.M. (n = 8). *P < 0.05, **P < 0.01 model group vs. the control group, ^#^P < 0.05, ^##^P < 0.01 drug delivery groups vs. the model group.

According to the one-way ANOVA, 5-HTP can significantly induce rat head twitches compared with the control group (52.5 ± 2.57, 2.71 ± 0.88, respectively, F(1,14) = 335.196, P = 0.000). As expected, aripiprazole and risperidone potently blocked 5-HTP-induced head twitches (31.5 ± 3.12, F(1,14) = 37.305, P = 0.01 and 9 ± 1.73, F(1,14) = 196.963, P = 0.000, respectively). PCC0104005 significantly inhibited head twitches induced by 5-HTP (20.17 ± 2.70, 24.86 ± 4.35, and 10.71 ± 4.81, respectively, F(3,28) = 29.990, P = 0.000) (Fig. 3). The effect of PCC0104005 was superior to aripiprazole, but only the group treated with 6.0 mg/kg PCC0104005 showed a significant difference compared with aripiprazole (F(1,14) = 19.083, P = 0.001) and have no significant difference compared to the control group. These data are consistent with the functional activity of PCC0104005 as a 5-HT_2A_ receptor antagonist *in vivo*.

### Effects of the acute administration of PCC0104005 on the learning and memory of MK-801-treated rats in the Morris water maze test

**Figure 4:**
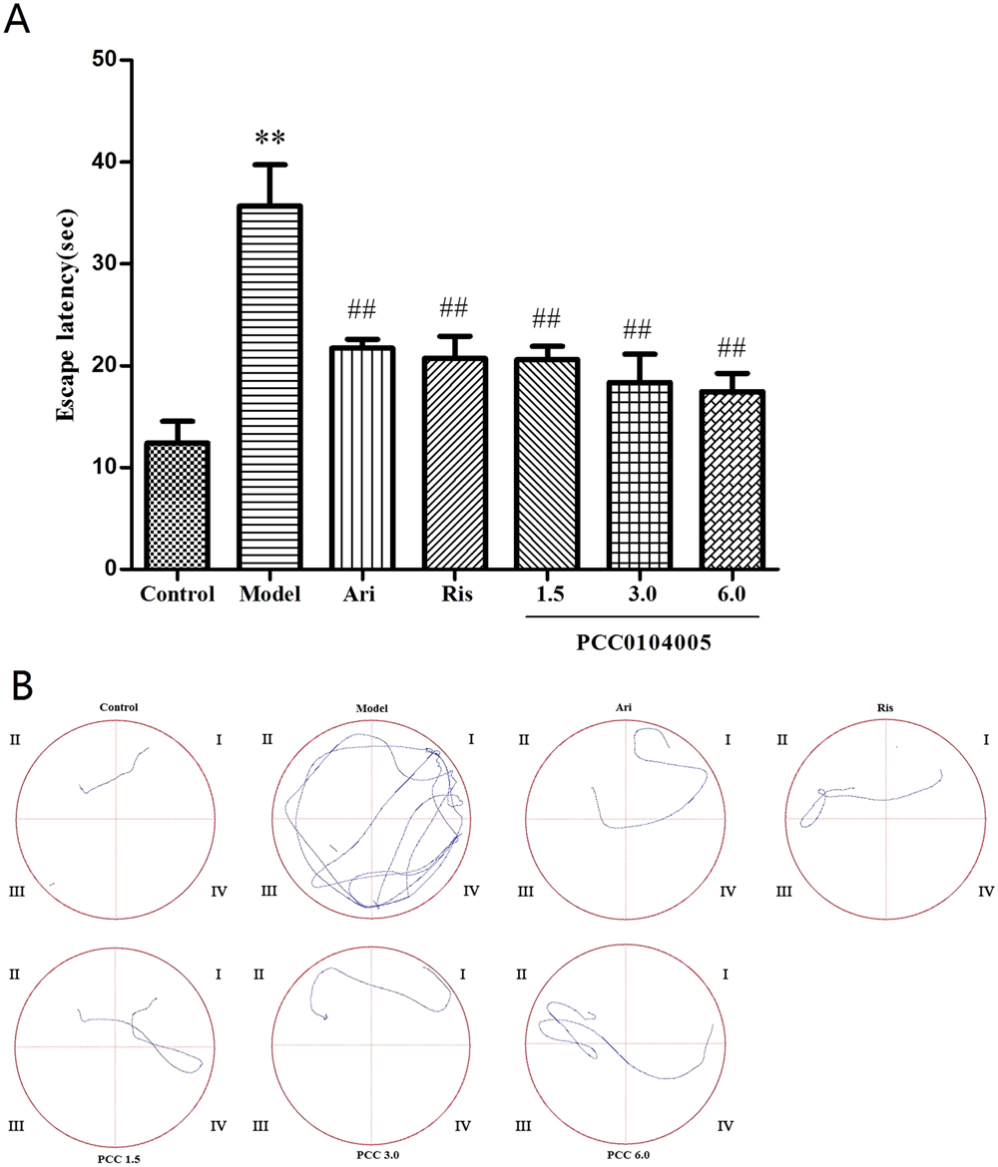
Effects of the acute administration of PCC0104005 on MK-801-treated rats in the MWM test. Rats were administered vehicle alone (0.5% methylcellulose, 1 ml/kg), PCC0104005 (1.5, 3.0, or 6.0 mg/kg), aripiprazole (Ari, 1.5 mg/kg, i.g.) or risperidone (Ris, 0.2 mg/kg) 1 hour before the experiment. Then, all animals were intraperitoneal injections of 0.9% saline or MK-801 (0.2 mg/kg, i.p.) 30 minutes before the test. (**A**) Effects of different drugs on the escape latency of rats. Results are presented as mean ± S.E.M. (n = 8). *P < 0.05, **P < 0.01 model group vs. the control group, ^#^P < 0.05, ^##^P < 0.01 drug delivery groups vs. the model group. (**B**) Representative swim paths during the probe test.

The Morris water maze is a preferred classic experiment that is widely used to study spatial learning and memory as correlates of brain function. Escape latency refers to the time the animal requires to locate the platform and is used to evaluate the animal’s learning and memory abilities. MK-801 significantly impaired learning and memory, as measured by the escape latency, compared to the control animals (35.67 ± 4.05 s and 12.38 ± 2.16 s, F(1,14) = 43.692, P = 0.000). As expected, aripiprazole and risperidone significantly decreased the escape latency (21.71 ± 0.86 s and 20.71 ± 2.18 s), suggesting that they exerted some beneficial effects on cognitive performance. The escape latencies of the 1.5, 3.0, 6.0 mg/kg PCC0104005 groups were significantly shorter than the model rats (20.60 ± 1.30 s, F(1,14) = 20.805, P = 0.002, 18.35 ± 2.76 s, F(1,14) = 22.318, P = 0.000, 17.45 ± 1.78 s, F(1,14) = 29.447, P = 0.000, respectively) (Fig. 4). PCC0104005 significantly decreased the escape latency, indicating that it improved spatial memory in SD rats treated with MK-801. A significant difference in the latency period was not observed among the PCC0104005 groups and aripiprazole group (F(3.28) = 2.117, P = 0.113).

### Effects of subchronic (sc) administration of PCC0104005 on the learning and memory of MK-801-treated rats in the Morris water maze test

**Figure 5:**
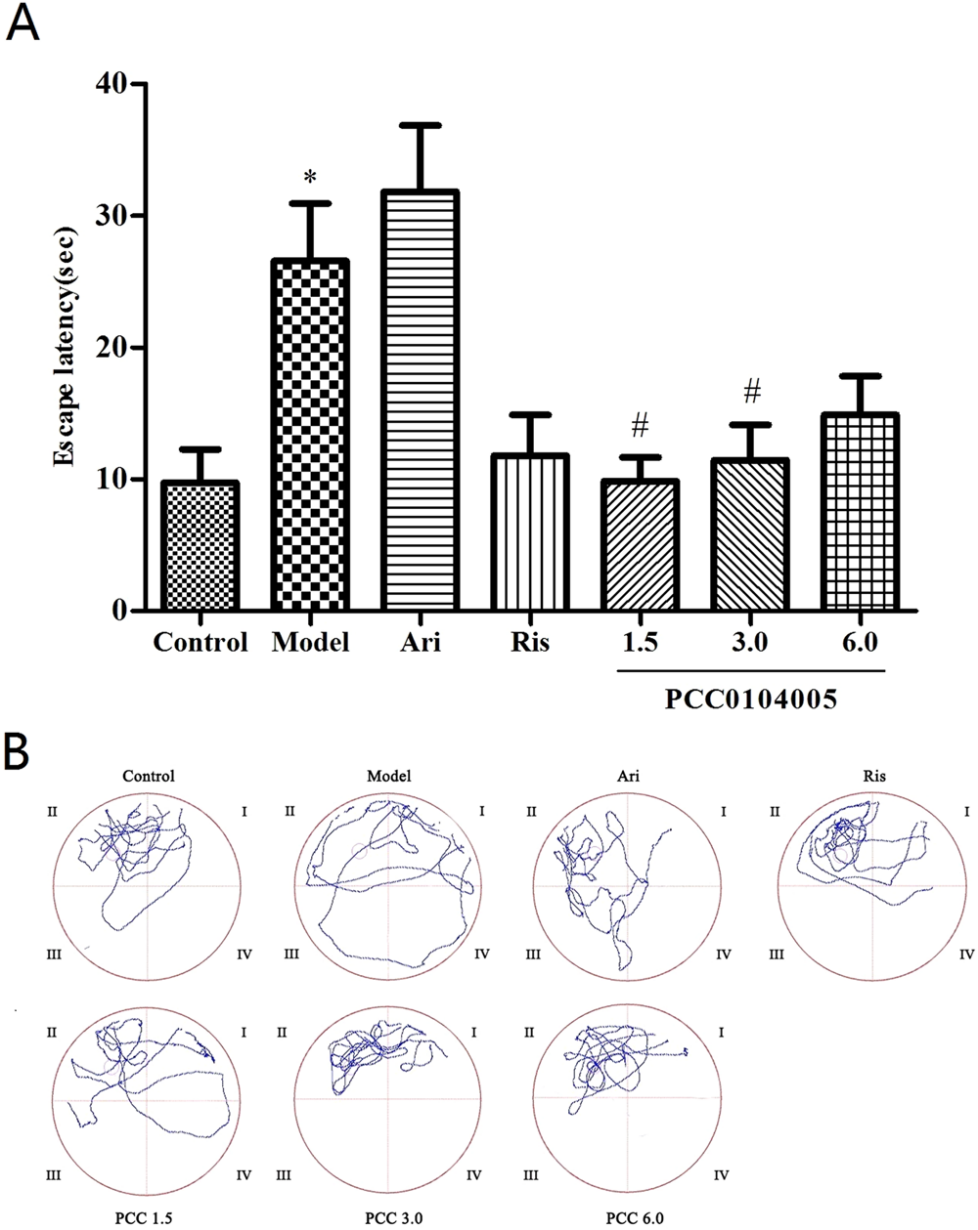
Effects of subchronic (sc) administration of PCC0104005 on MK-801-treated rats in the MWM test. Rats were administered an oral dose of vehicle alone or PCC0104005 (1.5, 3.0, or 6.0 mg/kg), aripiprazole (Ari, 1.5 mg/kg) or risperidone (Ris, 0.2 mg/kg) for 28 days. Beginning on the 15th day, intraperitoneal injections of 0.9% saline or MK-801 (0.3 mg/kg) were administered to 28th day. (**A**) Effects of different drugs on escape latency in rats. Results are presented as mean ± S.E.M. (n = 8). *P < 0.05, **P < 0.01 model group vs. the control group, ^#^P < 0.05, ^##^P < 0.01 drug delivery groups vs. the model group. (**B**) Representative track activity of rats in the target quadrant on the 6th day.

According to the one-way ANOVA, MK-801 significantly impaired learning and memory, as measured by the escape latency, compared to the control animals (25.56 ± 4.36 s and 9.75 ± 2.52 s, F(1,14) = 40.883, P = 0.002). As expected, risperidone significantly decreased the escape latency (11.81 ± 3.06 s, F(1,14) = 26.071, P = 0.006), suggesting that it exerted some beneficial effects on cognitive performance. However, the escape latency of aripiprazole-treated animals was significantly extended (31.83 ± 5.00 s) for as yet unknown reasons. The escape latencies of the 1.5, 3.0, 6.0 mg/kg PCC0104005 groups were significantly shorter than the model rats (9.85 ± 1.80 s, F(1,14) = 46.420, P = 0.000; 11.45 ± 2.70 s, F(1,14) = 30.781, P = 0.000; 14.88 ± 2.95 s, F(1,14) = 3.05, P = 0.036, respectively) (Fig. 5). Significant differences in the latency period were not observed among groups treated with risperidone and the three doses of PCC0104005 (P > 0.05). Moreover, a significant difference in the latency period was not observed among PCC0104005 groups (P > 0.05).

### Novel object recognition test

As early as 1950, Berlyne observed that rats spent significantly more time exploring a novel object than two familiar objects. Subsequently, the novel object recognition (NOR) task was developed, based on the natural propensity of rats to explore novel objects. It is a non-rewarding, ethologically relevant, relatively simple test. Such tests are increasingly being used to study and screen potential novel antipsychotic drugs. Indeed, the NOR task has been listed under the TURNS initiative as relevant for studying visual learning and memory deficits in schizophrenia (TURNS.ucla.edu).

**Figure 6:**
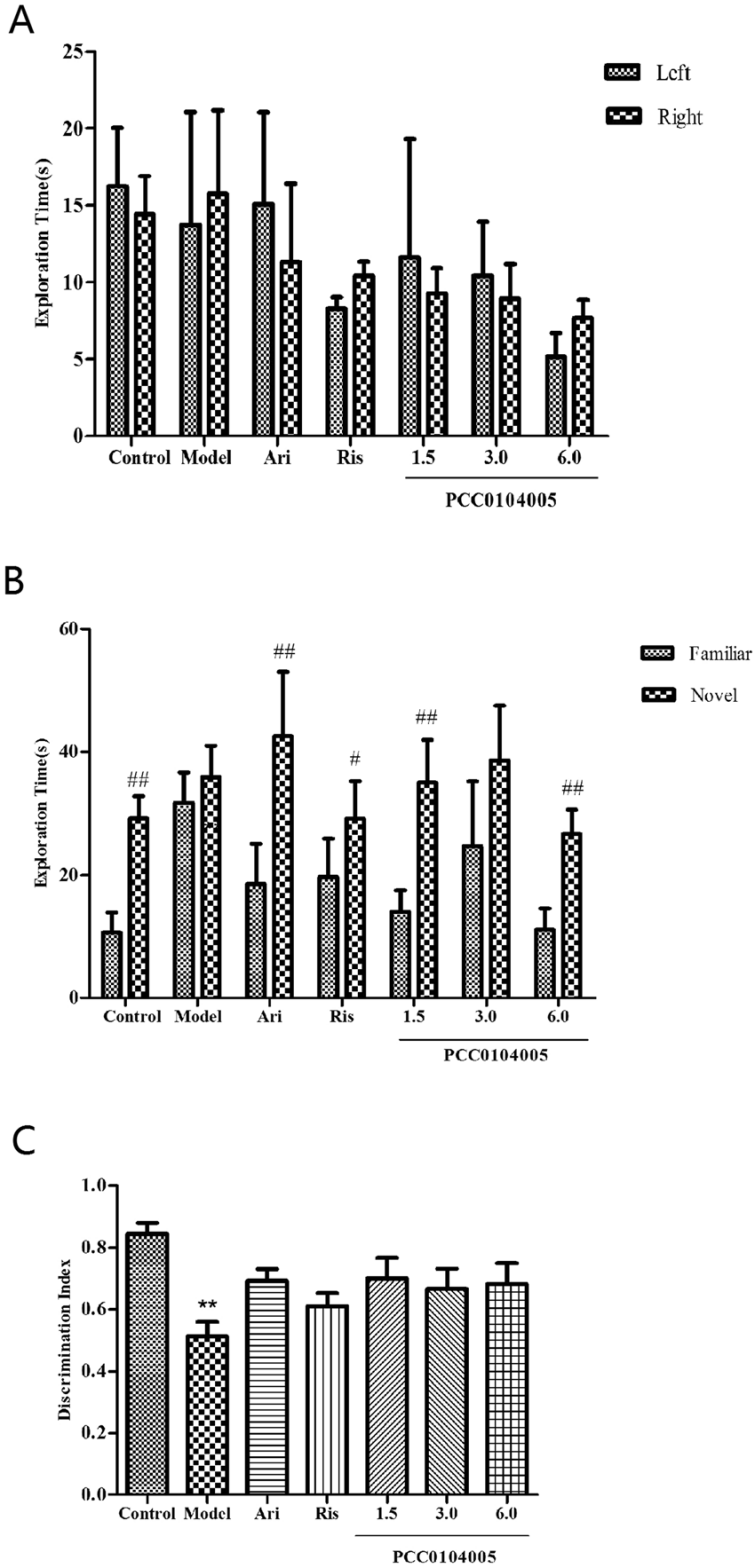
The effect of PCC0104005 on MK-801-induced NOR deficit in rats. SD rats were administered an oral dose of vehicle alone PCC0104005 (1.5, 3.0, or 6.0 mg/kg), aripiprazole (Ari, 1.5 mg/kg) or risperidone (Ris, 0.2 mg/kg) for 28 days. Beginning on the 15th day, intraperitoneal injections of 0.9% saline or MK-801 (0.3 mg/kg) to 28th day. On the day of testing, each rat was placed in the NOR chamber and exposed to two identical objects (Left and Right) for a period of 10 min (Acquisition Trial). Rats were then returned to their home cage for an inter-trial interval (ITI) of 1.5 h, the entire box was cleaned, and both objects removed. One was replaced with an identical familiar copy and one was replaced with a novel object. Following the ITI, rats were returned to the apparatus and allowed to explore the familiar and novel objects in the test box during a 10-min retention trial (Retention Trial). (**A**) Acquisition Trial: A preference for the left or the right object was not observed. (**B**) Retention Trial: With the exception of the model group and 3.0 mg/kg PCC0104005 group, the other groups showed significant increases in the exploration of novel object compared to the familiar object (^#^P < 0.05, ^##^P < 0.01). Data are expressed as means ± SEM (n = 10 per group). (**C**) Discrimination Index: All drug administration groups exhibited an increase in the MK-801-induced reduction in DI. ^##^P < 0.01: significant reduction in DI compared to the control group.

Two-way ANOVA did not show a significant effect of the treatment group or a difference in the time spent exploring two identical objects during the Acquisition Trial of the NOR task. A paired t test confirmed the lack of discrimination between the identical objects in all groups (Fig. 6A). During the 10-min Retention Trial, two-way ANOVA revealed a significant discrimination between the familiar and novel object (P < 0.01) and a significant effect of the drug treatment groups (P < 0.01). The paired sample t test did not reveal a significant difference in the time the MK-801-treated rats spent exploring the novel and the familiar objects in the Retention Trial. Compared with the model group, drug-treated groups displayed a significant increase in the time spent exploring the novel object compared with the familiar object (Fig. 6). One-way ANOVA revealed a significant difference in DI between the model group and control group (0.51 ± 0.04 and 0.84 ± 0.03, P = 0.01). PCC0104005, aripiprazole and risperidone improved the effects.

### Measurement of forelimb catalepsy in mice

**Figure 7:**
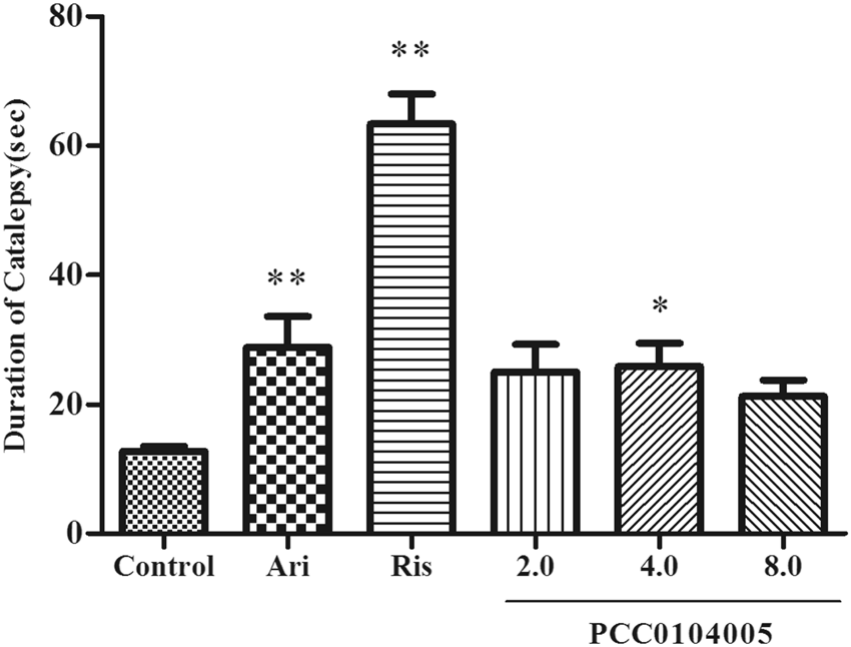
The effect of PCC0104005 on catalepsy duration in mice. Mice were administered an oral dose of vehicle alone (0.5% methylcellulose in water, 1 ml/kg) or PCC0104005 (2.0, 4.0, or 8.0 mg/kg), aripiprazole (2.0 mg/kg) or risperidone (Ris, 0.25 mg/kg)1 h before the test. The longest duration of catalepsy in each mouse was recorded. Results are presented as mean ± S.E.M. (n = 8). *P < 0.05, **P < 0.01 drug delivery groups vs. the control group.

We tested the compound for the induction of forelimb catalepsy in mice to further examine the potential for PCC0104005 to induce motor side effects. Mice receiving oral aripiprazole and risperidone treatments showed obvious forelimb catalepsy compared to the control mice (28.84 ± 4.72 s, F(1,14) = 0.657, P = 0.008 and 63.69 ± 4.57 s, F(1,14) = 99.27, P = 0.000). Mice in the 4.0 mg/kg PCC0104005 group displayed a statistically significant increase in forelimb catalepsy compared with the control group (25.89 ± 3.59 s and 12.77 ± 0.75 s, F(1,14) = 7.357, P = 0.017) as measured in the grip test (Fig. 7). In contrast, mice in the 2.0 mg/kg and 8.0 mg/kg PCC0104005 groups did not exhibit significant forelimb catalepsy (22.56 ± 2.65 s and 21.23 ± 2.53 s), suggesting that PCC0104005 does not increase the risk of catalepsy. In addition, mice treated with PCC0104005 left the steel rod within a significantly shorter time than aripiprazole-treated mice (P < 0.01).

### Effects on plasma prolactin, total cholesterol or triglyceride levels in rats

**Figure 8:**
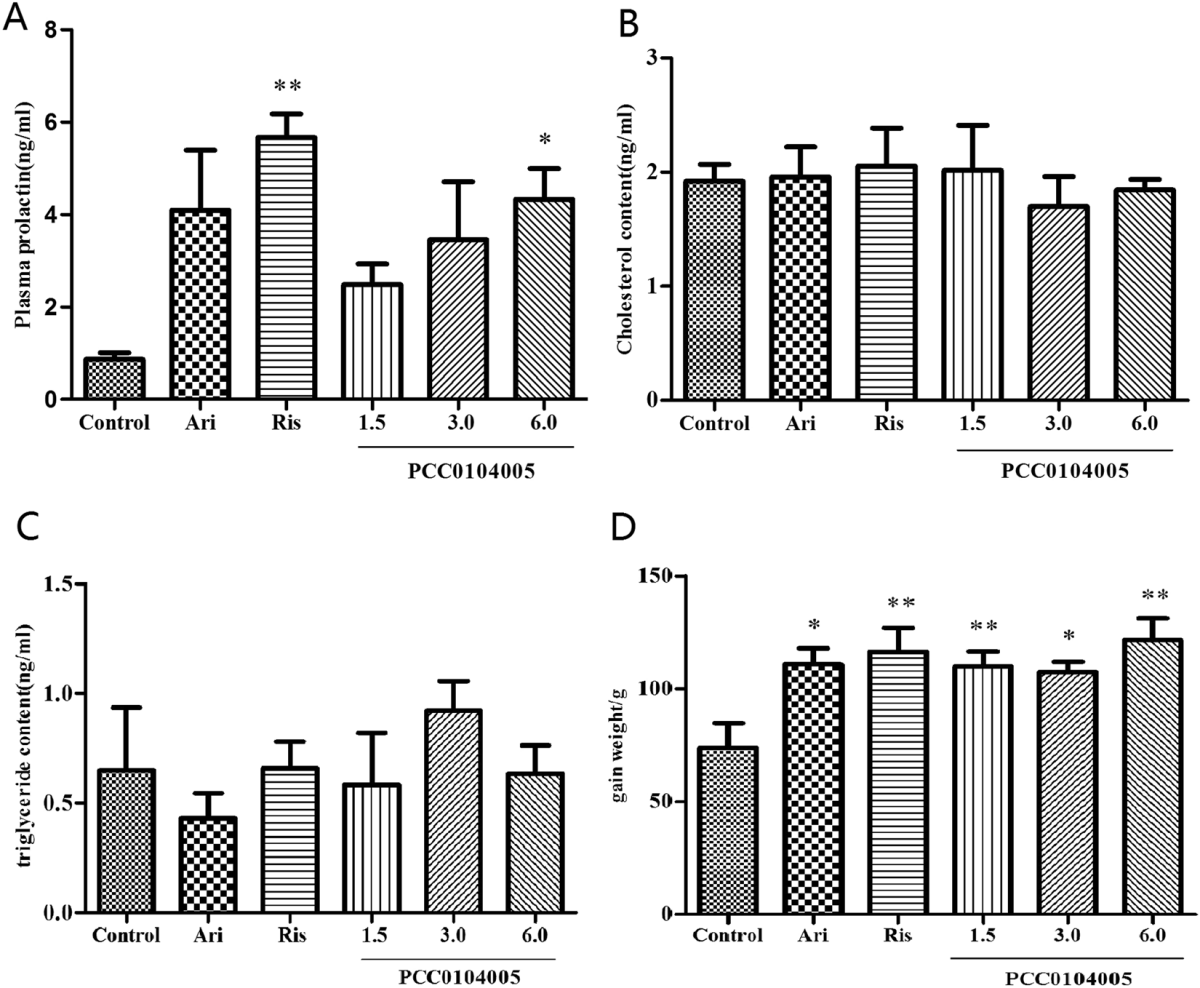
The adverse reactions that may be caused by PCC0104005. Rats were administered vehicle alone, PCC0104005 (1.5, 3.0, or 6.0 mg/kg), aripiprazole (Ari, 1.5 mg/kg, i.g.) or risperidone (Ris, 0.2 mg/kg) for 28 days before testing. Results are presented as mean ± S.E.M. (n = 3–5). (**A**) Plasma prolactin levels in different groups. Plasma prolactin levels were assessed using a commercially available Prolactin ELISA Kit. *P < 0.05, **P < 0.01 drug delivery groups vs. the control group. (**B**,**C**) Plasma total cholesterol and triglyceride levels in different groups. (**D**) Weight changes in different groups. *P < 0.05, **P < 0.01 drug delivery groups vs. the control group.

Plasma prolactin levels were assessed as described in detail by Cosi *et al*. (2006). Aripiprazole produced an increase in prolactin plasma levels, but the difference was not significant compared with the control group (4.09 ± 1.31 ng/ml and 0.869 ± 0.139 ng/ml, F(1,4) = 4.664, P = 0.139). Risperidone produced a significant increase in prolactin plasma levels (5.67 ± 0.51 ng/ml, F(1,4) = 56.877, P = 0.002), whereas only 6.0 mg/kg PCC0104005 significantly increased the plasma prolactin levels (4.32 ± 0.66 ng/ml, F(1,4) = 18.380, P = 0.013) (Fig. 8A). The 1.5 mg/kg and 3.0 mg/kg PCC0104005 treatments also increased the prolactin levels, but the difference was not significant compared with the control group (2.48 ± 0.77 ng/ml, F(1,4) = 5.601, P = 0.077 and 3.45 ± 2.17 ng/ml, F(1,4) = 3.038, P = 0.310). Compared with the control group, the body weights of rats in each experimental group were significantly increased (110.0 ± 6.54 g, 107.5 ± 4.43 g, 121.7 ± 9.54 g for 1.5, 3.0, and 6.0 mg/kg PCC0104005, respectively; 110.8 ± 7.12 g for aripiprazole and 116.4 ± 10.51 g for risperidone), but the differences among each treated group were not significant (p > 0.05). The plasma total cholesterol (TC) and triglyceride levels were not significantly different compared to the control group (P > 0.05) (Fig. 8B,C).

## Discussion

It is known that alterations in the function of the prefrontal cortex may contribute or even play a substantial role in disrupted flexibility observed in schizophrenia and its animal models^16^. It has been shown that MK-801 produces hyperlocomotion and perhaps most importantly a deficit in various cognitive domains^17,18^. Thus, the most probable explanation for the significant antagonism of the MK-801-induced locomotor hyperactivity is that PCC0104005 or aripiprazole block MK-801-evoked dopamine release. PCC0104005 is a D_2_ receptor partial agonist (Ki = 0.11 nM), consistent with current clinical studies^19^. The hyperactivity induced by MK-801 was decreased in PCC0104005-treated rats, consistent with the reduction in dopamine D_2_ receptor stimulation. The high affinity of PCC0104005 for the D_2_ receptor is speculated to exert beneficial effects on positive symptoms, but may be accompanied by an increased incidence of adverse effects, such as EPS and hyperprolactinemia. Simultaneously, the potential for D_2_ antagonist-like adverse effects (e.g., EPS and hyperprolactinemia) may be lower than other D_2_ receptor antagonist antipsychotics^6,9^. Although PCC0104005 has high affinity for the D_2_ receptor, it also shows fewer adverse reactions in animals, which may result in clinical advantages.

Antipsychotic drugs targeting dopamine D_2_ receptors may affect the positive symptoms of schizophrenia; however, drugs targeting non-DA D_2_ receptors (DA D_1_, D_3_ and D_4_), serotonin receptors (5-HT_2A_, 5-HT_1A_, 5-HT_3_, 5-HT_6_, 5-HT_7_), alpha-adrenergic receptors and other neurotransmitters are considered effective treatments for the negative symptoms of schizophrenia^20,21^. PCC0104005 has a moderate affinity for human 5-HT_2A_ receptors (Ki = 5.1 nM) and significantly inhibited the 5-HTP induced head twitch behaviour. 5-HTP is a precursor of 5-HT and is converted to 5-HT by degradation *in vivo*. The negative symptoms of schizophrenia may be associated with 5-HT_1A_ and 5-HT_2A_. Antagonism of 5-HT_2A_ receptors is the primary mechanism involved in head twitching. 5-HT_2A_ partial agonism slightly increased the release of substantia nigral and striatal DA to reduce the extrapyramidal response. 5-HT_2A_ partial agonism blocks the 5-HT_2A_ receptor in the prefrontal cortex, while increasing DA release and improving the negative symptoms^22^. At the same time, 5-HT_2A_ receptor partial agonistic effect is believed to contribute to antipsychotic activity, while reducing a variety of adverse reactions^9,22,23^.

The high affinity 5-HT_2A_/dopamine D_2_ receptor is associated with successful treatment of the negative symptoms of schizophrenia^24^, and these effects may be important for the relief of cognitive dysfunction. In previous studies, serotonin and adrenergic receptor-selective ligands (such as WAY-100635, M100907 and others) improved cognitive impairments in various NMDA receptor antagonist-induced animal models^24,25^. In our study, PCC0104005 and aripiprazole reversed MK-801-induced deficits, potentially by interacting with serotonin receptors and adrenoceptors^24^.

NMDAR antagonists cause rodent dysfunction and are the most widely used model for studying memory deficits associated with schizophrenia^26^. MK-801 has been shown to induce a deficit in acquisition in the Morris water maze, object recognition taskand other tests of relational and spatial memory including the active place avoidance task^16^. It is currently believed that the efficacy of atypical antipsychotics drugs, compared with antipsychotics drugs, in reversing the NOR deficit of NMDAR antagonists is thought to be explained by their greater affinity for 5-HT_2A_ over the dopamine D_2_ receptor^27^. It is generally considered that 5-HT agonism or antagonism contributes to the ability of antipsychotics at sub-D_2_ blocking doses to ameliorate cognitive impairments and negative symptoms in schizophrenia^27^. We have shown that NMDAR antagonist reduces parvalbumin expression in GABAergic interneurons in the frontal cortex, dentate gyrus and the CA2/3 region of the hippocampus^28,29^.

In our study, MK-801 increased the escape latency during the test period indicating that MK-801 impaired the reference spatial memory. Risperidone exhibited stronger blockade of dopamine D_2_ receptors compared to aripiprazole and PCC0104005, which may contribute to cognitive impairment observed in SD rats. Based on the results of our study, PCC0104005 significantly reduced the escape latency of rats and improved MK-801-induced memory impairments, but the differences were not significant compared to aripiprazole, consistent with the current clinical findings.

As expected, MK-801-induced recognition memory impairments were attenuated by PCC0104005. These data are completely consistent with the effects of other atypical and novel antipsychotics, such as clozapine, risperidone, and aripiprazole, on performance in this reversal learning task^30^. PCC0104005, the selective 5-HT_2A_ receptor partial agonism and the 5-HT_6_ receptor antagonist, attenuated the deficits in the NOR task induced by an NMDA receptor antagonist. PCC0104005 had a lower 5-HT_2A_ receptor occupancy at given D_2_ receptor occupancy, which may lead to clinical advantages. High affinity for and selectivity toward the dopamine D_3_ receptor distinguishes PCC0104005 from compounds like risperidone, which was reported to possess antipsychotic-like properties. Partial agonism at D_3_ receptors is involved in the antipsychotic and procognitive profiles, as well as effects on affective states^31,32^.

PCC0104005 did not significantly increase the plasma prolactin level, which may be related to the preferential affinity of PCC0104005 for dopamine D_2_ receptors than 5-HT_2A_ receptors, and the relative antagonistic activity toward the D_2_ receptor. This finding emphasizes the importance of the precise balance of DA D_2_ antagonism versus 5-HT_1A_ agonism for the optimal pharmacological activity of a new generation antipsychotics targeting both of these receptors, which requires further discussion^31^. PCC0104005 has fewer adverse reactions in animals, which may result in clinical advantages.

The consequences of the affinities of PCC0104005 for (and antagonist effects on) a_1A_ and a_1B_-adrenoceptors are more difficult to predict because of the lack of selective compounds for studying the functional importance of these receptors. Based on results obtained from genetically modified mice, a_1B_-antagonism may contribute to antipsychotic-like activity and effects on stimulant-induced reward^33^. Inhibition of peripheral a_1A_-adrenoceptors (for which brexpiprazole and aripiprazole have moderate affinities) is thought to be important for the regulation of blood pressure, but may not contribute to the overall effects^34^. PCC0104005 has high affinity for H_1_ and 5-HT_2C_ receptors and may have a tendency to cause side effects such as sedation and weight gain^34,35^.

The main findings are summarized below. (1) PCC0104005 induced behavioural effects consistent with dopamine D_2_ receptor blockade and antagonism at the 5-HT_2A_ receptor. (2) A well-balanced combination of dopamine receptors and serotonin receptors confers a favourable behavioural profile on PCC0104005, characterized by efficacy in models predictive of antipsychotic activity (dopamine D_2_ receptor partial agonism), together with a lack of cataleptogenic activity or serotonin syndrome induction (3) PCC0104005 alone had no detrimental effect on these locomotor hyperactivity model.

### Therapeutic potential of PCC0104005

PCC0104005 is non-inferior compared with similar anti-schizophrenic drugs such as aripiprazole and provides new drug candidates for the treatment of schizophrenia. Ultimately, the potential of PCC0104005 for the treatment of schizophrenia and other psychiatric and neurological disorders awaits further study in humans. The compound is currently under investigation in advanced human clinical studies.

## Materials and Methods

### Drugs

PCC0104005, a hydrochloric acid, was synthesized at ShanDong Luye Pharmaceutical Ltd. Aripiprazole was obtained from Otsuka Pharmaceutical Europe, Bristol-Myers Squibb Polska. Risperidone was obtained from Xian Janssen Pharmaceutical Ltd. MK-801 and 5-HTP was obtained from Sigma-Aldrich Chemical Co. (St. Louis, MO). All receptor binding studies were performed by Cerep Panlabs.

PCC0104005, aripiprazole and risperidone were dissolved in solution of 0.5% (w/v) methylcellulose (400 cP, #M0430, Sigma-Aldrich Chemical Co., Inc.) in saline. Oral dosing solutions were prepared fresh daily. MK-801 and 5-HTP were dissolved in physiological saline and protected from light.

### Animals

Male NIH mice (20–25 g) and Sprague-Dawley (SD) rats (180–220 g) obtained from Beijing Vital River Laboratory Animal Technology Co., Ltd. were used for behavioural experiments and measurements of adverse reactions. In all cases, animals were maintained under standard laboratory conditions on a 12-h light/dark cycle with food and water available ad libitum and a minimum of a 1-week acclimation period prior to experimentation. This acclimation period was established to reduce the potential stress and agitation associated with transportation and handling. All experiments were conducted in accordance with the guidelines of the Ministry of Health of PR China and the Animal Care Committee of China Medical University. The study protocol was approved by the Experimental Animal Research Committee of Yantai University.

### *In vitro* binding affinity

The affinity of compounds for the human dopamine D_2_, D_3_, D_4_, 5-HT_2A_, 5-HT_2B_, 5-HT_2C_, 5-HT_6_, 5-HT_7_, H_1_, α_1A_, and α_1B_ receptors in transfected HEK-293 cells was determined using a radioligand binding assay. Cell membrane homogenates were incubated in the absence or presence of the test compound in a buffer. Nonspecific binding was measured in the presence of the standard reference compound. Following incubation, samples were rapidly filtered under a vacuum through glass fibre filters (GF/B, Packard) presoaked with 0.3% PEI and rinsed several times with ice-cold 50 mM Tris-HCl using a 96-sample cell harvester (Unifilter, Packard). Filters were dried and then radioactivity was counted in a scintillation counter (Topcount, Packard) using a scintillation cocktail (Microscint 0, Packard). The results are expressed as percent inhibition of the specific binding of the radioligand in the control. The standard reference compound was tested in each experiment at several concentrations to obtain a competition curve from which its Ki was calculated.

### Inhibition of MK-801-induced hyperactivity

SD rats (200–250 g, 8 animals/group) were habituated to activity chambers (grey PVC boxes 60*60 cm wide and 45 cm deep) comprising the TopScan monitoring system (CleverSys Inc.) and were maintained in a quiet observation room under low light conditions for at least 1 h before recording their activities. Rats were administered an oral dose of vehicle alone (0.5% methylcellulose, 1 ml/kg), PCC0104005 (1.5, 3.0, or 6.0 mg/kg), aripiprazole (1.5 mg/kg) or risperidone (0.2 mg/kg) 1 h before test. Then, all animals were intraperitoneally injected with 0.9% saline or MK-801(0.3 mg/kg) 10 minutes before test. Animals were returned to the activity chambers after the intraperitoneal injection, and locomotor activity as quantitated as the distance travelled (mm) was recorded for 10 to 20 minutes using the TopScan monitoring system. At the end of each trial, the faecal boli were cleaned to avoid the potential influence of intramaze cues (odour trails, etc.) on the rats^36^.

### Determination of 5-HTP-induced head twitch

SD rats (200–250 g, 8 animals/ group) were housed in the test room for at least 1 h before the experiment. Rats were administered an oral dose of vehicle alone (0.5% methylcellulose, 1 ml/kg), PCC0104005 (1.5, 3.0, or 6.0 mg/kg), aripiprazole (1.5 mg/kg) or risperidone (0.2 mg/kg) 1 h before the test. Then, all animals were intraperitoneally injected with 0.9% saline or 5-HTP (320 mg/kg) 40 min before the test. The number of head twitches was recorded during a period of 40–70 min after intraperitoneal injection^37^.

### Effects of the acute administration on the learning and memory of MK-801-treated rats in the Morris water maze (MWM) test

SD rats (200–250 g, 8 animals/ group) were trained continuously until all rats found the platform within 30 sec, and the training time depended on the training results. On the test day, rats were administered an oral dose of vehicle alone (0.5% methylcellulose, 1 ml/kg), PCC0104005 (1.5, 3.0, or 6.0 mg/kg), aripiprazole (1.5 mg/kg) or risperidone (0.2 mg/kg) 1 h before the test. Then, all animals were intraperitoneal injections of 0.9% saline or MK-801 (0.2 mg/kg, i.p.) 30 minutes before the test. The test was started and the escape latency of rats was measured during the test in which platform was not withdrawn. A trial was deemed complete as soon as the rat had located platform or when 60 s had elapsed, whichever occurred first. The swimming activity of each rat was monitored by TopScan monitoring system. At the end of each trial, the maze was cleaned. The maze was rotated by clockwise after each training day to avoid potential intramaze cues (odour trails, etc.)^38^.

### Effects of the subchronic administration on the learning and memory of MK-801-treated rats in the MWM test

SD rats (200–250 g, 8 animals/ group) were administered an oral dose of vehicle alone (0.5% methylcellulose, 1 ml/kg), PCC0104005 (1.5, 3.0, or 6.0 mg/kg), aripiprazole (1.5 mg/kg) or risperidone (0.2 mg/kg) for 28 days. Beginning on the 15th day, rats were intraperitoneally injected with 0.9% saline or MK-801(0.3 mg/kg) to the 28th day. The MWM test was started after the last injection. Each rat was trained in 4 trials per day for 5 consecutive days. The escape latency to find the submerged escape platform in the water maze was recorded with the TopScan monitoring system. Each training session lasted for a maximum of 60 s. If the escape latency exceeded 60 s, it was recorded as 60 s and the rat was manually guided to the platform. On day 6, the platform was removed and each rat was allowed to swim freely for 60 s.

### Novel object recognition (NOR) test

SD rats (200–250 g, 8 animals/ group) were administered an oral dose of vehicle alone (0.5% methylcellulose, 1 ml/kg), PCC0104005 (1.5, 3.0, or 6.0 mg/kg), aripiprazole (1.5 mg/kg) or risperidone (0.2 mg/kg) for 28 days. Beginning on the 15th day, rats were intraperitoneally injected with 0.9% saline or MK-801 (0.3 mg/kg) to the 28th day. Rats were testing after the last administration. Following a 10-min habituation session on the day of testing, each rat was placed in the NOR chamber (grey PVC boxes 60*60 cm wide and 45 cm deep) and exposed to two identical objects (Left and Right) for a period of 10 min (Acquisition Trial). The objects used in this experiment were rectangular plastic cases. The heights of the objects were comparable (10 ± 2 cm), and they were heavy enough to ensure that they would not be displaced by the animals. Rats were then returned to their home cage for an inter-trial interval (ITI) of 1.5 h and the entire box was cleaned. Then one was replaced with an identical familiar copy and one was replaced with a novel object. Following the ITI, rats were returned to the apparatus and allowed to explore the familiar and novel objects in the test box for a 10-min retention trial (Retention Trial). All experiments were monitored by TopScan monitoring system^39^.

### Measurement of forelimb catalepsy in mice

Mice were administered an oral dose of vehicle alone (0.5% methylcellulose, 1 ml/kg), PCC0104005 (2.0, 4.0, or 8.0 mg/kg), aripiprazole (2.0 mg/kg) or risperidone (0.25 mg/kg) 1 h before the test. Then each mouse was positioned such that both front paws rested on a 0.4 mm diameter steel rod (covered with non-slippery tape) placed 3.5 cm above the surface of the bench. The period of time that each mouse maintained this position was recorded. The experiment was repeated at least 10 times. Finally, the longest period of catalepsy for each mouse was recorded^40^.

### Effects on plasma prolactin, total cholesterol (TC) or triglyceride levels in rats

Rats were administered an oral dose of vehicle alone (0.5% methylcellulose, 1 ml/kg), PCC0104005 (1.5, 3.0, or 6.0 mg/kg), aripiprazole (1.5 mg/kg) or risperidone (0.2 mg/kg) for 1 month. Plasma prolactin levels were assessed using a commercially available Prolactin ELISA (Mouse/Rat) purchased from GenWay, according to the manufacturer’s instructions. Plasma TC and triglyceride levels were assessed using a Roche automatic biochemical analyser Cobas c 311.

### Statistical analysis

All data are expressed as the mean ± S.E.M. Between-group differences were analysed with SPSS software using one-way analysis of variance (ANOVA) and the least significance difference (LSD) post hoc test. Data from the same animals evaluated on several occasions were analysed using between-within univariate or multivariate ANOVA for repeated measures. Exploration data (in the acquisition and retention trials) in Novel object recognition test were analyzed by a two-way analysis of variance (ANOVA) followed by the pair-wise comparison when a significant effect was detected by the ANOVA. DI data was analyzed by one-way ANOVA followed by post hoc Bonferroni test when a significant effect was detected by ANOVA.
